# Differential Prognostic Associations of Monocyte-Containing and Traditional Systemic Inflammatory Indices with Overall Survival After Resection or Palliative Bypass for Pancreatic Cancer: A Retrospective Cohort Study

**DOI:** 10.3390/diseases14100358

**Published:** 2026-09-28

**Authors:** Rares Voda, Sohaib Ahmed, Ovidiu Aurelian Budișcă, Cătălin-Dumitru Cosma, Ovidiu Simion Cotoi, Călin Molnar, Vladimir Bacârea

**Affiliations:** 1 Faculty of Medicine, George Emil Palade University of Medicine, Pharmacy, Sciences and Technology of Târgu Mureș, 540139 Târgu-Mureș, Romania; rares.voda@umfst.ro (R.V.); ovidiu.budisca@umfst.ro (O.A.B.); ovidiu.cotoi@umfst.ro (O.S.C.); calin.molnar@umfst.ro (C.M.); vladimir.bacarea@umfst.ro (V.B.); 2 General Surgery Clinic No. 1, County Emergency Clinical Hospital of Târgu-Mureș, 540136 Târgu-Mureș, Romania; 3 Surgery Department, RCSI University of Medicine and Health Sciences, D09 YD60 Dublin, Ireland; sohaib.ahmd@outlook.com; 4 Clinical Pathology Department, Mures Clinical County Hospital, 540011 Târgu-Mureș, Romania; 5 Pathophysiology Department, George Emil Palade University of Medicine, Pharmacy, Sciences and Technology of Targu Mures, 540142 Târgu-Mureș, Romania

**Keywords:** pancreatic cancer, systemic inflammation, aggregate index of systemic inflammation, systemic inflammation response index, palliative bypass, pancreatic resection, overall survival, prognostic biomarkers

## Abstract

Background/Objectives: Systemic inflammatory indices may differ prognostically between resectable and palliative pancreatic cancer. We compared associations of the aggregate index of systemic inflammation (AISI), systemic inflammation response index (SIRI), neutrophil-to-lymphocyte ratio (NLR), systemic immune-inflammation index (SII), and monocyte-to-lymphocyte ratio (MLR) with overall survival after pancreatic resection or palliative bypass. Methods: This single-center retrospective cohort included 240 patients treated during 2013–2023 (99 resections; 141 palliative bypasses). Indices derived from preoperative blood counts were natural-log transformed, standardized to 1 SD, and entered separately into operative-group-stratified Cox models. Group-specific associations and interactions were estimated. Reconstructed AJCC eighth-edition stage was incorporated only in the resection stratum. Interaction *p* values were Holm-adjusted. AISI was designated primary and SIRI key secondary. Results: Overall, 215 deaths occurred. Median overall survival was 17.9 months after resection and 5.4 months after palliative bypass. AISI was associated with mortality following bypass (adjusted hazard ratio [HR] per 1-SD increase in ln(AISI), 1.54; 95% confidence interval [CI], 1.28–1.86) but not following resection (HR, 0.97; 95% CI, 0.75–1.25; interaction *p* = 0.003; Holm-adjusted *p* = 0.010). SIRI showed corresponding HRs of 1.60 (95% CI, 1.30–1.96) and 0.93 (95% CI, 0.72–1.20), respectively (interaction *p* = 0.001; Holm-adjusted *p* = 0.004). Exploratory MLR analysis also showed statistical heterogeneity. SII was associated with mortality following bypass, but its operative-group interaction was not significant. The conventional NLR interaction was also nonsignificant, although model-assumption violations limited its interpretation. The AISI and SIRI findings remained robust in the sensitivity analyses. Conclusions: AISI and SIRI showed statistical heterogeneity in their associations with overall survival between operative groups, with higher values associated with mortality following palliative bypass. These findings do not establish that the operative procedure modifies the underlying biological relationship. The absence of pathological staging in the bypass group leaves potential residual confounding by disease extent. Independent external validation is required before clinical implementation.

## 1. Introduction

Pancreatic cancer remains one of the most lethal malignancies worldwide. According to GLOBOCAN 2022, approximately 511,000 new cases and 467,000 deaths were recorded globally, illustrating the close correspondence between incidence and mortality [1]. Although survival has gradually improved, the overall five-year survival rate remains approximately 12%, with outcomes varying markedly according to the stage at diagnosis [2]. Clinical management depends on disease extent, with pancreatic cancer classified as resectable, borderline resectable, locally advanced, or metastatic; patient condition also informs treatment selection [3]. Even after potentially curative pancreatectomy, recurrence remains frequent. In a large cohort of patients undergoing resection for pancreatic ductal adenocarcinoma (PDAC), 76.7% developed recurrence, with a median recurrence-free survival of 11.7 months [4]. These findings underscore the need for accessible biomarkers capable of improving prognostic stratification across different clinical and operative settings.

Systemic inflammation is increasingly recognized as an important component of pancreatic cancer progression. Peripheral blood cell counts provide inexpensive and routinely available measures of the balance between tumor-promoting inflammation and antitumor immune competence. Among these biomarkers, the neutrophil-to-lymphocyte ratio (NLR) has been investigated most extensively. Elevated preoperative NLR was initially associated with reduced disease-free survival following curative resection of pancreatic adenocarcinoma [5]. A subsequent meta-analysis confirmed an association between elevated NLR and poorer survival after pancreatic cancer resection, although the thresholds applied across the included studies varied considerably [6]. Comparative studies also suggested that NLR may provide stronger prognostic information than the platelet-to-lymphocyte ratio (PLR) in resected PDAC [7]. The derived NLR, calculated without requiring the absolute lymphocyte count, has similarly undergone external validation in a large pancreatic cancer cohort [8].

More complex indices have subsequently been developed to integrate several components of the systemic inflammatory response. The systemic immune-inflammation index (SII), calculated using platelet, neutrophil, and lymphocyte counts, was associated with poorer overall survival among patients undergoing pancreatic cancer resection [9]. Its potential prognostic value has also been examined specifically following pancreaticoduodenectomy for PDAC [10]. However, the biological information captured by NLR and SII is predominantly centered on neutrophils, lymphocytes, and platelets and does not fully account for the potential contribution of circulating monocytes.

Monocytes are biologically relevant to pancreatic cancer because they can be recruited into the tumor microenvironment through the C–C motif chemokine ligand 2/C–C chemokine receptor type 2 axis and subsequently differentiate into tumor-associated macrophages [11]. These macrophages may promote immunosuppression, angiogenesis, stromal remodeling, local invasion, and metastatic dissemination, although their biological functions are heterogeneous and context-dependent [12]. This mechanistic rationale has stimulated interest in monocyte-containing inflammatory indices. A systematic review and meta-analysis demonstrated a prognostic association between the lymphocyte-to-monocyte ratio and survival in pancreatic cancer [13]. Other investigations comparing systemic inflammatory biomarkers have shown that composite measures incorporating inflammatory and nutritional parameters may also provide prognostic information [14].

The systemic inflammation response index (SIRI), which integrates neutrophil, monocyte, and lymphocyte counts, represents a potentially more comprehensive measure of the interaction between systemic inflammation and host immunity. SIRI has been associated with survival and oncological outcomes in patients undergoing pancreatic resection after neoadjuvant chemotherapy [15]. Other monocyte-containing measures, including the neutrophil-to-monocyte ratio, have also been proposed as prognostic markers in pancreatic cancer [16]. Nevertheless, these studies differed substantially regarding patient selection, disease stage, treatment setting, outcome definition, statistical adjustment, and the methods used to determine biomarker thresholds.

Importantly, the direction and magnitude of reported associations have not been consistent. Although most studies associated elevated inflammatory indices with poorer outcomes, a recent pancreaticoduodenectomy cohort reported longer survival among patients with higher NLR and SII values [17]. This apparently paradoxical result emphasizes the potential influence of case selection, obstructive jaundice, perioperative inflammation, treatment exposure, disease stage, and data-dependent cutoff selection. Consequently, inflammatory indices should not be regarded as universally applicable prognostic biomarkers without direct comparison and adjustment for relevant clinicopathological characteristics.

The aggregate index of systemic inflammation (AISI) extends the composite biomarker concept by incorporating neutrophil, monocyte, platelet, and lymphocyte count within a single index. Elevated AISI has demonstrated prognostic associations in hepatocellular carcinoma [18] and in patients undergoing surgery for non-small-cell lung cancer [19]. However, evidence regarding AISI in pancreatic cancer remains limited, and its prognostic performance has not been adequately compared with both traditional and monocyte-containing inflammatory indices across clinically distinct operative populations.

Most previous studies evaluated inflammatory indices separately in either resected or advanced pancreatic cancer and have frequently dichotomized these indices using cohort-specific thresholds. Such dichotomization may reduce statistical information, produce unstable effect estimates, and limit external reproducibility. Patients undergoing pancreatic resection and those requiring palliative bypass also represent clinically distinct populations with respect to disease extent, resectability, physiological status, and treatment intent. Pooling these populations under a single common biomarker effect, without formally assessing heterogeneity, may obscure group-specific associations and introduce confounding by disease severity. A valid comparative analysis should therefore retain the two operative groups as distinct strata and directly test whether biomarker–survival associations differ between them.

Accordingly, this retrospective cohort study evaluated the associations of baseline AISI, SIRI, NLR, SII, and the monocyte-to-lymphocyte ratio (MLR) with overall survival within the pancreatic-resection and palliative-bypass groups using a common comparative framework while estimating group-specific prognostic associations and formally testing biomarker-by-operative-group interactions. AISI was designated as the primary index, SIRI as the key secondary index, and NLR, SII, and MLR as exploratory comparators. All indices were analyzed as continuous, natural-log-transformed variables to avoid reliance on sample-specific thresholds. Cox models were stratified by operative group, thereby allowing different baseline mortality hazards in the two populations, and biomarker associations were estimated separately for each group. AJCC eighth-edition stage reconstructed from pT, pN, and documented M status was incorporated into the resection-group analyses. Because comparable pathological staging was unavailable following palliative bypass, stage was neither imputed nor included in that stratum.

## 2. Materials and Methods

### 2.1. Study Design, Setting, and Ethical Approval

This retrospective, single-center cohort study was conducted in the Department of Surgery of the Clinical County Emergency Hospital Târgu Mureș, Romania. The source population comprised patients who underwent surgery for suspected pancreatic malignancy between 1 January 2013 and 31 December 2023. Follow-up information was available through 9 April 2026. For patients undergoing pancreatic resection, pathological T (pT) and N (pN) categories and documented distant-metastasis (M) status were harmonized, and stage groups were reconstructed according to the eighth edition of the American Joint Committee on Cancer (AJCC) staging system [20]. Patients undergoing palliative bypass were not assigned a pathological AJCC stage because no pancreatic resection specimen was available. The study is reported in accordance with the Strengthening the Reporting of Observational Studies in Epidemiology (STROBE) recommendations [21]. The study was conducted in accordance with the Declaration of Helsinki and was approved by the Medical Ethics Committee for Clinical Drug Studies of the Târgu Mureș County Emergency Clinical Hospital (Decision No. Ad 23356, dated 10 September 2025). The analytical dataset was de-identified, and study results are reported exclusively in aggregate form.

### 2.2. Study Population and Cohort Construction

The institutional source database contained 245 patients who underwent surgery for suspected pancreatic malignancy during the study period. Eligibility was determined through a review of the available histopathological or cytological findings, cross-sectional imaging, operative reports, and hospital records. In the pancreatic-resection group, diagnostic classification was based primarily on final histopathological assessment. In the palliative-bypass group, pancreatic malignancy was histologically or cytologically confirmed when tissue sampling was available; otherwise, the diagnosis was based on concordant radiological, operative, and clinical findings and was considered clinically presumed pancreatic cancer. Five patients with confirmed competing histological diagnoses were excluded following clinicopathological review: two with pancreatic neuroendocrine tumors, two with ampullary adenocarcinoma, and one with pancreatic squamous-cell carcinoma. The final analytical cohort therefore comprised 240 patients, including 99 who underwent pancreatic resection and 141 who underwent palliative biliary and/or gastrointestinal bypass without tumor resection. Because the study represented a retrospective census of all eligible patients treated during the study period, no formal prospective sample-size calculation was performed. The available analytical dataset did not permit reliable enumeration of palliative-bypass patients with histological or cytological confirmation. Consequently, the number and percentage with tissue-confirmed disease could not be reported.

### 2.3. Data Collection and Operative Groups

Demographic, clinical, laboratory, operative, pathological, and survival information was retrospectively extracted from institutional records. Baseline variables included age at operation, sex, calendar year of surgery, type of operation, total serum bilirubin concentration, complete blood-count parameters, pathological findings, and survival status. Total serum bilirubin was recorded in mg/dL, whereas absolute blood-cell counts were recorded in ×10^9^/L. Patients were classified into two clinically distinct operative groups according to the operative report. The resection group included pancreaticoduodenectomy, distal or subtotal pancreatectomy, total pancreatectomy, and other oncological pancreatic resections. The palliative-bypass group included patients who underwent biliary and/or gastrointestinal bypass without resection of the pancreatic tumor. These procedures were grouped together because they shared a palliative operative intent without pancreatic tumor resection, consistent with the study’s comparison of resection and palliative-bypass populations. This grouping included biliary bypass, gastrointestinal bypass, and combined procedures; it did not imply equivalence of their clinical indications or prognostic profiles. The analyses did not separately estimate inflammatory-index associations by bypass type. Exploratory procedures and biopsy-only operations were not included in either group. Operative-group classification was based on the procedure completed during the index operation and was not subsequently modified according to reconstructed AJCC stage. Eight patients with documented M1 disease therefore remained in the resection group because pancreatic resection had been performed. The available analytical dataset did not permit reliable description of the clinical circumstances leading to resection in these patients, their metastatic sites, or whether metastasectomy or another combined procedure was performed.

The available analytical dataset did not contain sufficiently complete information on CA19-9, performance status, nutritional status, biliary drainage, cholangitis or other active infections, resection-margin status, or neoadjuvant and adjuvant chemotherapy or radiotherapy to permit their consistent inclusion in the multivariable models. These variables could not be supplemented for the present analysis, and sensitivity analyses incorporating them were not performed. Histological grade was available for only a subset of resection patients and was excluded from the multivariable models because of extensive missingness. The available treatment information did not permit reliable reporting of the proportions receiving neoadjuvant or adjuvant chemotherapy or radiotherapy in each operative group, or analyses stratified by treatment exposure. Missing treatment information was not interpreted as indicating that treatment had not been administered.

### 2.4. Pathological Assessment and AJCC Staging

For resected tumors, pathological T (pT) and N (pN) categories were abstracted from the final histopathological report. Distant-metastasis status was determined from the available pathological, operative, and radiological documentation and recorded as M0 or M1. AJCC eighth-edition stage groups were reconstructed from the recorded pT, pN, and M categories, including for patients operated on before the formal implementation of this edition [20]. This reconstruction resolved two internally inconsistent manually entered stage labels by assigning the stage indicated by the recorded pT, pN, and M categories. Complete pT, pN, and M information was available for all 99 patients in the resection group, permitting reconstruction of the AJCC eighth-edition stage group.

For the primary stage-adjusted analysis, AJCC stage was grouped as stages I–II versus stages III–IV to limit the number of model parameters relative to the number of observed deaths. Sensitivity analyses represented stage as an ordinal variable from IA to IV, and separately used the individual T4, node-positive, and M1 components. A comparable pathological stage was not available for patients undergoing palliative bypass because these patients did not have a resection specimen. Pathological stage was therefore neither imputed nor included in models for this group. The available analytical dataset did not contain clinical or radiological stage, metastatic status, tumor size, or vascular involvement for the palliative-bypass group in a form permitting additional adjustment for disease extent.

Histological grade was available for 30 out of the 99 resection patients (30.3%): 25 had moderately differentiated (G2) and five had poorly differentiated (G3) tumors. Grade was not recorded in the available analytical dataset for the remaining 69 patients. The reason for its absence could not be reliably established; missingness in the dataset does not necessarily indicate that grade was absent from the original histopathological reports. No grade-adjusted analysis restricted to patients with recorded grade was performed.

### 2.5. Inflammatory Indices

Preoperative absolute neutrophil, lymphocyte, monocyte, and platelet counts were obtained from the routine blood sample collected closest to, and before, the index operation. A single measurement per patient was used to calculate all inflammatory indices. The indices were recalculated directly from the component blood-cell counts rather than using previously derived spreadsheet values. Calculations used the numerical values of the absolute blood-cell counts expressed in ×10^9^/L.

The following indices were calculated:

- Neutrophil-to-lymphocyte ratio (NLR) = neutrophils/lymphocytes;
- Systemic immune-inflammation index (SII) = platelets × neutrophils/lymphocytes;
- Monocyte-to-lymphocyte ratio (MLR) = monocytes/lymphocytes;
- Systemic inflammation response index (SIRI) = neutrophils × monocytes/lymphocytes;
- Aggregate index of systemic inflammation (AISI) = neutrophils × monocytes × platelets/lymphocytes.

Calculations used the numerical values of the absolute blood-cell counts expressed in ×10^9^/L. AISI was designated as the primary inflammatory index, SIRI as the key secondary index, and NLR, SII, and MLR as exploratory comparators. Values with a missing or non-positive component required for a particular formula were considered unavailable for that index. No laboratory values or derived indices were imputed.

### 2.6. Outcome and Follow-Up

Overall survival was the sole endpoint evaluated. Information on recurrence and cause of death was unavailable for this analysis; therefore, recurrence-free and cancer-specific survival could not be assessed. Overall survival was defined as the interval from the date of the index operation to death from any cause. Patients without a recorded death were censored on the date of their last documented follow-up. Follow-up information available after the end of the surgical inclusion period was retained, with the latest recorded follow-up occurring on 9 April 2026. No fixed-time-point survival endpoint, conventional diagnostic receiver-operating-characteristic analysis, or data-derived biomarker cutoff was used. This decision avoided loss of information from dichotomizing continuous biomarkers and reduced the risk of optimistic, sample-specific threshold selection.

### 2.7. Statistical Analysis

Continuous variables were summarized as medians and interquartile ranges because several laboratory measurements and inflammatory indices were markedly right-skewed. Categorical variables were summarized as frequencies and percentages. Baseline characteristics were described separately for the resection and palliative-bypass groups. Between-group baseline testing was not used to imply comparability because the operative groups represented different clinical treatment pathways rather than randomized exposures. Overall survival was estimated using the Kaplan–Meier method. Survival curves for the two operative groups were compared descriptively using the log-rank test. Associations between inflammatory indices and overall survival were examined using Cox proportional-hazards regression. Because the inflammatory indices were right-skewed, each was transformed using the natural logarithm. For each index, log-transformed values were standardized using the mean and population standard deviation calculated across all patients with a valid value for that index in the combined cohort. This pooled scale was applied to both operative groups so that their hazard ratios represented the same numerical increase in the log-transformed index. Group-specific standardization would instead express each hazard ratio per a different-sized increment when the within-group standard deviations differ, complicating direct comparison of the association estimates. Hazard ratios are therefore reported per 1 pooled SD increase in the natural-log-transformed index, with 95% confidence intervals. This common scaling does not remove differences in index distributions or clinical characteristics between operative groups. Each inflammatory index was entered into a separate model because the five indices share several hematological components and their simultaneous inclusion would introduce substantial mathematical collinearity.

The primary analysis used a joint Cox model stratified by operative group, thereby permitting separate baseline hazard functions for the resection and palliative-bypass groups. The model allowed group-specific coefficients for each standardized log-transformed inflammatory index and for age, sex, ln (total bilirubin), and calendar year. Age, ln(total bilirubin), and calendar year were standardized using the available valid observations from the overall cohort before model fitting. For each index, heterogeneity was evaluated using a likelihood-ratio test comparing a model with a common biomarker coefficient against a model with separate coefficients for the two operative groups. AJCC stages III–IV versus I–II was incorporated only into the resection stratum; pathological stage was neither imputed nor modeled in the palliative-bypass stratum. Adjusted hazard ratios were therefore reported separately for each operative group. Sensitivity analyses within the resection group modeled AJCC stage ordinally, and separately used the individual pathological T, N, and M components.

Missing information was handled using complete-case analysis separately for each marker-specific model. The number of patients and deaths contributing to each model is reported with the corresponding results. No outcome, staging, laboratory, or covariate values were imputed. The proportional-hazards assumption was evaluated using likelihood-ratio tests comparing adjusted models with and without marker-by-log (1 + follow-up time) interaction terms. Marker-specific tests evaluated the inflammatory index, whereas the global tests jointly evaluated time interactions for all model coefficients. Follow-up time was expressed in days. Tied event times were handled using the Breslow approximation. Apparent Harrell concordance indices were calculated as exploratory measures of model discrimination and were not interpreted as evidence of validated prognostic performance or clinical utility. The analyses evaluated associations with overall survival and did not assess whether the indices predicted differential benefit from treatment.

AISI was designated as the primary inflammatory index, SIRI as the key secondary index, and NLR, SII, and MLR as exploratory comparators. To address multiplicity, Holm-adjusted *p* values were calculated within each family of five marker-specific tests, including the five operative-group interaction tests and the five tests conducted within each operative subgroup. All tests were two-sided. For test families subjected to multiplicity correction, statistical significance was defined as a Holm-adjusted *p* value < 0.05. Effect estimates and their 95% confidence intervals were emphasized rather than isolated significance testing. Statistical analyses were performed using Python version 3.12.13 (Python Software Foundation, Fredericksburg, VA, USA), pandas version 2.2.3, NumPy version 2.3.5, and SciPy version 1.17.0. Figures were generated using Matplotlib version 3.10.8.

### 2.8. Use of Generative Artificial Intelligence-Assisted Tools

OpenAI ChatGPT and Codex (OpenAI, San Francisco, CA, USA) were used during manuscript preparation to assist with the development and review of statistical-analysis code, verification and presentation of numerical outputs, drafting and English-language editing of the manuscript text as well as the formatting and visual presentation of tables and figures. These tools did not independently determine patient eligibility, alter source clinical data, assign pathological classifications or AJCC stage, or make final scientific or clinical decisions. All data transformations, statistical code and outputs, interpretations, and AI-assisted text were critically reviewed and approved by the authors, who take full responsibility for the accuracy, originality, validity, and integrity of the manuscript.

## 3. Results

### 3.1. Cohort Construction and Follow-Up

Among the 245 patients assessed for eligibility, five were excluded following clinicopathological review: two patients with pancreatic neuroendocrine tumors, two with ampullary adenocarcinoma, and one with pancreatic squamous-cell carcinoma. The final analytical cohort therefore comprised 240 patients: 99 who underwent pancreatic resection and 141 who underwent palliative biliary and/or gastrointestinal bypass without tumor resection (Figure 1).

During follow-up, 215 deaths occurred, while 25 patients were censored. Complete pT, pN, and documented M information was available for all 99 patients in the resection group, permitting reconstruction of the AJCC eighth-edition stage group. Missingness was limited for the baseline hematological variables and calculated inflammatory indices, although histological grade was unavailable for 69 out of the 99 resection patients. Missing data were not imputed, and each marker-specific model used complete cases for its required variables (Table S1). Of the 240 eligible patients, the primary AISI, SIRI, and MLR models each included 227 patients with 206 deaths, the NLR model included 234 patients with 210 deaths, and the SII model included 233 patients with 210 deaths. Thus, 13 patients (5.4%) were excluded from each AISI, SIRI, and MLR model compared with six (2.5%) from the NLR model and seven (2.9%) from the SII model.

### 3.2. Baseline, Operative, and Pathological Characteristics

The median age of the study population was 66 years (IQR, 61–73 years), and 137 patients (57.1%) were male (Table 1). The baseline clinical profiles differed descriptively between the operative groups. Compared with patients who underwent pancreatic resection, those undergoing palliative bypass were older (median, 69 vs. 65 years) and more frequently admitted as emergencies (37.6% vs. 7.1%). Patients undergoing palliative bypass also had higher recorded total bilirubin values (median, 9.47 vs. 1.01), higher neutrophil counts (5.93 vs. 4.93), and lower lymphocyte counts (1.28 vs. 1.60). Consistent with these hematological differences, all five inflammatory indices were descriptively higher in the palliative-bypass group. Median AISI was 636 versus 453, SIRI was 2.70 versus 1.80, MLR was 0.50 versus 0.37, NLR was 4.60 versus 3.10, and SII was 1052 versus 789. These distributions demonstrate the substantially different clinical case mix of the two operative groups and support the subsequent estimation of biomarker associations separately within each group (Table 1).

Within the resection group, pancreaticoduodenectomy was the most frequently performed procedure (74/99, 74.7%), followed by distal or subtotal pancreatectomy (23/99, 23.2%; Table 2). All resections were performed using an open approach. Most tumors were classified as pT3 (62/99, 62.6%), and 61 patients (61.6%) had pathological nodal involvement. AJCC eighth-edition stage IIB was the most frequent stage group (37/99, 37.4%), while 24 patients (24.2%) had stage III or IV disease. Complete pathological T, N, and M information was available for all 99 resection patients. Histological grade was unavailable for 69 patients (69.7%) and was therefore not included in the multivariable survival models (Table 2).

### 3.3. Overall Survival According to Operative Group

At the data cutoff of 9 April 2026, the median follow-up estimated using the reverse Kaplan–Meier method was 114.6 months (95% CI, 96.8–136.1 months), and the maximum observed follow-up was 149.6 months. Overall survival was summarized separately for the two operative groups because they represented clinically distinct disease and treatment contexts. Kaplan–Meier estimates differed markedly according to operative group (log-rank *p* < 0.001; Figure 2). Median overall survival was 17.9 months (95% CI, 12.1–26.4 months) following pancreatic resection and 5.4 months (95% CI, 4.3–6.7 months) following palliative bypass. The estimated 12-month overall survival probability was 60.6% (95% CI, 50.3–69.4%) in the resection group and 22.7% (95% CI, 16.2–29.9%) in the palliative-bypass group. At 24 months, the corresponding survival probabilities were 44.4% (95% CI, 34.5–53.9%) and 10.6% (95% CI, 6.2–16.4%), respectively. Because operative allocation reflected clinical selection and disease resectability rather than random assignment, this unadjusted survival comparison should be interpreted descriptively and not as an estimate of the causal effect of pancreatic resection.

### 3.4. Operative-Group-Specific Prognostic Associations and Robustness Analyses

In the prespecified stratified Cox analysis, the association between AISI and overall survival differed significantly according to operative group (likelihood-ratio interaction *p* = 0.003; Holm-adjusted *p* = 0.010; Table 3 and Figure 3). Among patients undergoing palliative bypass, each 1-SD increase in ln(AISI) was associated with a 54% higher adjusted hazard of death (adjusted HR, 1.54; 95% CI, 1.28–1.86; *p* < 0.001). No corresponding association was identified following pancreatic resection (adjusted HR, 0.97; 95% CI, 0.75–1.25; *p* = 0.788). SIRI demonstrated a similar operative-group-specific pattern. Each 1-SD increase in ln(SIRI) was associated with a higher mortality hazard following palliative bypass (adjusted HR, 1.60; 95% CI, 1.30–1.96; *p* < 0.001), whereas the estimate remained close to the null following pancreatic resection (adjusted HR, 0.93; 95% CI, 0.72–1.20; *p* = 0.600). The SIRI-by-operative-group interaction remained significant after multiplicity correction (raw *p* = 0.001; Holm-adjusted *p* = 0.004).

In the exploratory MLR analysis, higher values were associated with mortality following palliative bypass (adjusted HR, 1.67; 95% CI, 1.33–2.09; *p* < 0.001) but not following resection (adjusted HR, 0.89; 95% CI, 0.69–1.14; *p* = 0.353). The MLR-by-operative-group interaction remained significant after Holm correction (adjusted *p* = 0.001). Despite this statistical significance, the MLR findings are hypothesis-generating because MLR was designated an exploratory comparator. SII was associated with mortality within the palliative-bypass group (adjusted HR, 1.24; 95% CI, 1.05–1.45; raw *p* = 0.010; subgroup Holm-adjusted *p* = 0.021); however, the SII-by-operative-group interaction was not significant (raw *p* = 0.126; Holm-adjusted *p* = 0.251). Under the conventional linear proportional-hazards specification, neither the NLR coefficient within either operative group nor the NLR-by-operative-group interaction reached statistical significance (interaction raw *p* = 0.235; Holm-adjusted *p* = 0.251). However, diagnostic analyses identified both nonlinearity and time dependence in the palliative-bypass group. These conventional model results therefore do not establish the absence of an NLR–survival association or exclude differences between operative groups. Adding AISI to the corresponding base model yielded an apparent increase in Harrell’s C-index from 0.570 to 0.653 following palliative bypass (ΔC = 0.083) and from 0.672 to 0.677 following resection (ΔC = 0.005). The corresponding apparent increases for SIRI were 0.072 and 0.007, respectively (Table 4, Panel A). These estimates were calculated in the corresponding index-specific complete-case samples and were not corrected for optimism. The numerical differences do not establish superiority of one index over another.

The absence of an association between AISI or SIRI and survival following resection remained unchanged when AJCC stage was modeled as an ordinal variable, when pathological T4, node-positive, and M1 categories were entered separately, and when the analysis was restricted to patients with AJCC stages I–III (Table 4, Panel B). In all of these analyses, the 95% confidence intervals included unity. After additional adjustment for emergency presentation and local-invasion status, with age, sex, ln(total bilirubin), and calendar year retained in the models, higher AISI and SIRI remained associated with mortality in the palliative-bypass group. The adjusted HRs per 1-SD increase in the natural-log-transformed index were 1.51 (95% CI, 1.25–1.83) for AISI and 1.57 (95% CI, 1.28–1.92) for SIRI. The corresponding operative-group interactions remained significant for AISI (raw *p* = 0.009; Holm-adjusted *p* = 0.027) and SIRI (raw *p* = 0.003; Holm-adjusted *p* = 0.010). Similar findings were obtained in the 30-day landmark analysis, with Holm-adjusted interaction *p* values of 0.017 for AISI and 0.016 for SIRI. In both robustness analyses, the estimates remained close to unity following pancreatic resection and remained adverse following palliative bypass (Table 4).

Hazard ratios represent the relative mortality hazard per 1 pooled SD increase in the natural-log-transformed index. For each index, the SD was calculated across all patients with a valid index value in the combined cohort; the same numerical increment therefore applies to both operative groups. This unit is not a group-specific SD or a one-unit increase in the untransformed index. Each index was entered into a separate Cox model. Models were stratified by operative group and allowed group-specific effects for age, sex, ln(total bilirubin), and calendar year. AJCC stages III–IV versus I–II was included only in the resection stratum because pathological staging was unavailable following palliative bypass. Ties were handled using the Breslow method. Within-group *p* values are Wald tests. Interaction *p* values are likelihood-ratio tests comparing models with common versus operative-group-specific biomarker coefficients. Holm correction was applied across the five marker-specific interaction tests. AISI was the primary index, SIRI was the key secondary index, and NLR, SII, and MLR were exploratory (Table 3).

### 3.5. Model Diagnostics and Functional-Form Assessment

The diagnostic analyses provided no evidence of a departure from proportional hazards or linearity on the log-hazard scale for AISI or SIRI in either operative group (all marker-specific diagnostic *p* ≥ 0.258; Table S3). The corresponding global proportional-hazards tests were also nonsignificant. These findings supported the use of single adjusted hazard ratios to summarize the associations of AISI and SIRI with overall survival within each operative group. No significant assumption violations were detected for any inflammatory index in the resection group. Similarly, MLR and SII showed no statistically significant evidence of nonlinearity or time dependence following palliative bypass, although the marker-specific proportional-hazards test for SII approached the conventional significance threshold (*p* = 0.068).

In the palliative-bypass group, diagnostic tests identified nonlinearity in the NLR–mortality association (*p* < 0.001) and evidence that the association varied over follow-up (*p* = 0.007). Consequently, the conventional linear proportional-hazards estimate in Table 3 (HR, 1.14; 95% CI, 0.98–1.33) is an inadequate summary of this association. It is retained for transparency but should not be interpreted as a constant hazard ratio across NLR values or follow-up times, or as evidence that NLR lacks prognostic relevance.

The restricted cubic spline analysis suggested a nonmonotonic relationship, with wider confidence intervals at the sparsely represented extremes of the NLR distribution (Figure S2). This analysis assessed functional form but did not resolve the detected time dependence. The spline and time-interaction analyses were diagnostic assessments; a prognostic model jointly accommodating nonlinear and time-varying NLR associations was not developed. Accordingly, these analyses do not provide clinically interpretable time-specific NLR effects or support a clinical threshold.

The index-specific proportional-hazards assessment was based on a likelihood-ratio test comparing models with and without a marker-by-log(1 + follow-up time) interaction. The global proportional-hazards test jointly assessed time interactions for all model coefficients. Nonlinearity was assessed by comparing the linear index specification with a four-knot restricted cubic spline model, with knots positioned at the 5th, 35th, 65th, and 95th percentiles. Models were adjusted for age, sex, ln(total bilirubin), and calendar year; resection models were additionally adjusted for AJCC stages III–IV versus I–II. Diagnostic *p* values were not multiplicity-adjusted. A small *p* value indicates evidence against the corresponding model assumption. AISI, aggregate index of systemic inflammation; MLR, monocyte-to-lymphocyte ratio; NLR, neutrophil-to-lymphocyte ratio; SII, systemic immune-inflammation index; SIRI, systemic inflammation response index.

## 4. Discussion

This retrospective cohort study identified marked operative-group heterogeneity in the associations between systemic inflammatory indices and overall survival in patients undergoing surgery for pancreatic cancer. Three principal findings emerged. First, higher AISI, the prespecified primary index, was associated with a substantially higher mortality hazard following palliative bypass but not following pancreatic resection. The AISI-by-operative-group interaction remained significant after Holm correction and across expanded-adjustment and 30-day landmark analyses. Second, SIRI demonstrated an almost identical pattern, with an adverse association following palliative bypass and an estimate close to unity following resection. Third, the exploratory MLR analysis showed similar heterogeneity, whereas neither NLR nor SII demonstrated a statistically significant operative-group interaction. The significant AISI and SIRI interactions indicate statistical heterogeneity of the estimated prognostic associations between the observed operative groups. They do not establish that undergoing resection or palliative bypass biologically modifies these associations.

The operative-group interaction requires careful clinical interpretation. Patients undergoing palliative bypass were older, more frequently admitted as emergencies, had substantially higher bilirubin and inflammatory-index values, and had considerably shorter survival than patients undergoing pancreatic resection. Operative strategy therefore represented not only a surgical procedure, but also a marker of different disease extent, physiological condition, and clinical presentation. Although the stratified Cox models allowed separate baseline hazards and group-specific covariate effects, and the expanded models additionally accounted for emergency presentation and local-invasion status, residual differences between the groups inevitably remain. Accordingly, the observed interactions demonstrate statistical heterogeneity of the biomarker–survival associations but do not establish that operative strategy itself biologically modifies these relationships.

The survival difference between the operative groups was expected but should not be interpreted causally. Resection was undertaken in patients considered suitable for tumor removal, whereas palliative bypass was performed in patients with unresectable or otherwise non-resectional disease. The median overall survival of 17.9 months following resection remained consistent with the aggressive natural history and high recurrence burden of pancreatic malignancy even after potentially curative surgery [3,4]. Conversely, the median survival of 5.4 months following palliative bypass reflects the unfavorable clinical context of this group. The Kaplan–Meier comparison consequently describes two clinically selected populations and does not estimate the therapeutic effect of resection versus bypass.

The absence of a significant association between the inflammatory indices and survival after resection contrasts with several previous reports. Elevated preoperative NLR has been associated with reduced disease-free and overall survival after pancreatic cancer resection, including in dedicated meta-analyses [5,6]. SII has also been reported as an adverse prognostic marker in patients undergoing pancreatic resection or pancreaticoduodenectomy [9,10], while assessments combining preoperative and postoperative inflammatory parameters have suggested that changes in these indices may carry additional prognostic information [22]. However, the literature is notably inconsistent. Pointer et al. found NLR, but not PLR or LMR, to be associated with survival following resection of early-stage pancreatic ductal adenocarcinoma [23]. In contrast, Gupta et al. identified no significant prognostic association for NLR, PLR, or LMR [24], and Chawla et al. reported that neither NLR nor PLR predicted survival in resectable disease [25]. More recently, Kazlow et al. reported the counterintuitive finding that higher NLR and SII were associated with better survival following pancreaticoduodenectomy [17].

The present resection results therefore fall within an already heterogeneous evidence base. Importantly, pathological TNM categories were complete in the resection group, and the absence of AISI and SIRI associations persisted when stage was represented as AJCC III–IV versus I–II, as an ordinal variable, or through separate T4, node-positive, and M1 components. Restriction to stages I–III also produced estimates below unity with confidence intervals that included the null. Nevertheless, these analyses should not be interpreted as demonstrating equivalence or definitive absence of an inflammatory contribution. The primary resection models included 94 patients and 74 deaths for AISI and SIRI, and their confidence intervals remained compatible with moderately protective or adverse associations. Limited statistical precision, clinical heterogeneity, perioperative influences on blood counts, and incomplete information on other established prognostic factors may all have attenuated the observed relationships.

The associations observed for monocyte-containing indices provide a rationale for further investigation but do not demonstrate a specific biological mechanism. Experimental and translational studies have implicated the CCL2/CCR2 axis in inflammatory monocyte recruitment to the pancreatic tumor microenvironment and subsequent differentiation into tumor-associated macrophages [11]. These macrophages may contribute to immunosuppression, stromal remodeling, angiogenesis, invasion, and therapeutic resistance [12]. This literature provides biological context rather than evidence that the same pathway explains the present findings. Our study measured peripheral blood-cell counts and did not assess CCL2/CCR2 signaling, monocyte subsets, or tumor-associated macrophage abundance or function. Associations reported between lymphocyte-to-monocyte ratio, survival, and obstructive jaundice further support considering both tumor-related and systemic clinical influences when interpreting monocyte-containing indices [13,26,27].

SIRI has previously demonstrated prognostic associations after pancreatic resection and neoadjuvant chemotherapy [15], and inflammatory markers incorporating monocytes have also been investigated in advanced pancreatic cancer treated with FOLFIRINOX [28]. In the present cohort, SIRI was associated with mortality following palliative bypass, whereas no statistically significant association was observed following resection. Higher inflammatory indices may reflect a combination of tumor burden, biliary obstruction, infection or cholangitis, tissue injury, impaired nutritional reserve, and declining performance status. In the present analysis, the associations of AISI and SIRI with mortality following palliative bypass persisted after adjustment for ln(total bilirubin) and additional adjustment for emergency presentation and local-invasion status. However, these adjustments do not exclude residual confounding associated with biliary obstruction or acute systemic illness. Information on infection or cholangitis was insufficient to permit direct adjustment or an analysis excluding affected patients. Accordingly, these indices should be interpreted as nonspecific markers of systemic inflammation and clinical condition; the present findings do not establish that their associations arise specifically from tumor-related inflammation.

AISI was selected as the primary index because it integrates neutrophil-, monocyte-, platelet-, and lymphocyte-related components. Its association following palliative bypass was robust, with an adjusted 54% increase in mortality hazard per 1-SD increase in ln(AISI). Although AISI has been investigated in hepatocellular and lung malignancies [18,19], direct evidence in pancreatic cancer remains limited. The present study therefore provides an initial pancreatic-cancer-specific signal rather than validation of an established biomarker.

AISI was not shown to be superior to SIRI or the other inflammatory indices. AISI and SIRI were strongly correlated (Spearman ρ = 0.94), and correlations among the five indices ranged from 0.60 to 0.94 (Table S2 and Figure S1). These indices should therefore be regarded as overlapping measures of systemic inflammation derived from shared hematological components. Their separate associations with survival do not establish that any index provides independent or incremental prognostic information beyond the others. The designation of AISI as the primary index and SIRI as the key secondary index reflects the analytical hierarchy rather than demonstrated comparative superiority. Likewise, the exploratory MLR association does not identify a causal role for circulating monocytes or establish that monocyte-containing indices provide incremental prognostic information beyond conventional markers.

The SII findings illustrate the distinction between an association within one operative group and evidence of a difference between groups. Higher SII was associated with mortality following palliative bypass, but the SII-by-operative-group interaction was not significant (Holm-adjusted *p* = 0.251). Consequently, these findings do not establish that the SII–survival association differs between resection and palliative bypass. Statistical significance in one group and nonsignificance in the other is not sufficient evidence of heterogeneity; conversely, the nonsignificant interaction does not establish equivalence between groups. Although the MLR-by-operative-group interaction remained significant after multiplicity correction, MLR was designated an exploratory comparator. Its findings therefore carry less evidential weight than the primary AISI and key secondary SIRI analyses. Multiplicity correction does not change this exploratory status. The MLR results should be regarded as hypothesis-generating and require confirmation in an independent cohort with a prespecified MLR analysis.

NLR presented a separate methodological issue. Its linear effect was not significant in either operative group, but diagnostic testing identified both nonlinearity and time dependence following palliative bypass. Consequently, the single proportional-hazards estimate cannot adequately summarize its association. This finding may partially explain the inconsistent direction and magnitude of NLR results across previous studies, particularly when different cohorts use different data-derived cut-offs [6,17,23,24,25]. Dichotomization can conceal nonlinear relationships, create threshold instability, and produce apparently contradictory results across populations. The spline analysis in the present study was therefore used to demonstrate model misspecification rather than to propose a new NLR threshold. No clinically actionable cut-off can be derived from these data.

The apparent discrimination results were consistent with the operative-group-specific associations. Adding AISI or SIRI produced minimal changes in Harrell’s C-index following resection but larger apparent increases following palliative bypass. These apparent improvements in discrimination should be considered exploratory. The models were fitted and evaluated in the same samples, without bootstrap optimism correction or external validation, so their performance in new patients remains uncertain. Calibration was not assessed; therefore, agreement between the model-estimated survival probabilities and observed outcomes was not evaluated. Clinical net benefit was also not assessed. Accordingly, the observed C-index changes do not establish a validated improvement in prognostic performance or justify clinical implementation.

The potential clinical attraction of these indices lies in their availability, low cost, and derivation from routinely obtained complete blood counts. If externally validated, AISI or SIRI could potentially contribute to research-oriented risk stratification among patients undergoing palliative bypass, including adjustment or stratification in clinical studies and the identification of patients requiring intensified supportive care. At present, however, neither index should influence assessments of resectability, selection of an operative procedure, systemic treatment, or prognosis communicated to individual patients. Their incremental value over established clinical variables has not yet been demonstrated.

This study has several strengths. The final cohort included 240 patients and 215 deaths, providing a high overall event proportion and prolonged follow-up. Inflammatory indices were recalculated from their absolute hematological components rather than relying on previously recorded ratios. Right-skewed indices were natural-log transformed and standardized, allowing hazard ratios to be expressed on a comparable 1-SD scale. Pathological TNM information was complete in all resection patients, permitting reconstruction of AJCC eighth-edition stage and several alternative stage-adjustment analyses. The analysis formally tested biomarker-by-operative-group interactions instead of inferring heterogeneity from significance in one subgroup and nonsignificance in another. Multiplicity was addressed using Holm correction, and robustness was examined through expanded adjustment, alternative stage specifications, restriction to stages I–III, and a 30-day landmark analysis. Finally, proportional-hazards and functional-form assumptions were evaluated explicitly, identifying the inadequacy of a conventional linear NLR model following palliative bypass.

Several limitations must also be acknowledged. First, the retrospective, single-center design introduces the possibility of selection bias, measurement variability, and residual confounding and restricts generalizability. Second, reconstructed AJCC eighth-edition stage based on pT, pN, and documented M status was available only in the resection group. The palliative-bypass group lacked comparable pathological staging, and the available dataset did not permit additional adjustment using clinical or radiological stage, metastatic status, tumor size, or vascular involvement. Consequently, residual confounding by tumor burden and disease severity may account for part of the observed associations in this group and the differences between operative groups. These findings represent prognostic associations and do not establish causal effects of the inflammatory indices or the operative procedure. Furthermore, histopathological confirmation was not uniformly available in the palliative-bypass group. For patients without tissue confirmation, eligibility relied on integrated radiological, operative, and clinical evidence; some misclassification of the primary tumor site or histological subtype therefore cannot be excluded.

Third, adjustment for other clinically relevant prognostic factors was limited by data availability. Histological grade was unavailable in 69.7% of resection patients, preventing its inclusion in the primary models without substantial loss of observations. Residual confounding by tumor differentiation therefore remains possible, and patients with recorded grade may differ systematically from those without it. A sensitivity analysis restricted to patients with available grade was not performed. Such an analysis would involve at most 30 patients, including only five with G3 tumors, raising concerns about precision and model stability. Information on CA19-9, performance status, nutritional status, biliary drainage, cholangitis or other active infections, resection-margin status, and neoadjuvant or adjuvant treatment was insufficient for consistent inclusion in the models or additional sensitivity analyses. The estimated associations may therefore remain confounded by differences in clinical condition, biliary obstruction or infection, tumor characteristics, and treatment exposure.

Fourth, the indices were calculated from a single preoperative blood measurement selected as the closest available sample before surgery. This analysis did not assess within-patient variability or longitudinal changes in inflammatory indices. The analysis also did not distinguish samples obtained before versus after biliary drainage or assess their timing relative to antibiotic treatment or hospitalization. These circumstances may have influenced the measured inflammatory indices. Insufficient information on active infection or cholangitis prevented a sensitivity analysis excluding patients with these conditions. Although the expanded models accounted for emergency presentation, this adjustment does not eliminate potential confounding related to the clinical context of blood collection. The findings therefore concern the selected preoperative measurement and should not be interpreted as evidence of a persistent inflammatory state. A single measurement may capture transient inflammation related to the clinical circumstances surrounding surgery. Furthermore, insufficient information on active infection or cholangitis prevented a sensitivity analysis excluding patients with these conditions. The 2013–2023 inclusion period also introduces potential variation in treatment practices over time; adjustment for calendar year cannot substitute for patient-level information on treatment regimens, timing, and receipt. The findings therefore concern the selected preoperative measurement and should not be interpreted as evidence of a persistent inflammatory state. Fifth, complete-case analysis resulted in different analytical samples across the marker-specific models, with 2.5–5.4% of the eligible cohort excluded from the primary analyses. Although these proportions were relatively small, they do not establish that the exclusions were unrelated to clinical characteristics or survival. No comparison of the included and excluded patients or multiple-imputation sensitivity analysis was performed. Selection bias therefore cannot be excluded, and differences between marker-specific results may partly reflect differences in sample composition. Sixth, overall survival captures death from any cause and may reflect the combined influence of cancer progression, comorbidities, postoperative complications, and subsequent treatment. Recurrence-free and cancer-specific survival could not be evaluated, and subsequent oncological treatment was not sufficiently documented to assess its contribution. The observed associations therefore cannot be attributed specifically to cancer progression or cancer-related mortality. Although the 30-day landmark analysis assessed the sensitivity of the findings to early postoperative deaths, it did not isolate the effects of postoperative complications, later non-cancer mortality, or subsequent treatment. Finally, although multiplicity correction was applied, several analyses remained exploratory, and the strong mathematical correlation among the indices limits claims regarding the comparative or independent biological importance of any individual marker. The palliative-bypass group also included biliary, gastrointestinal, and combined bypass procedures, which may represent different patterns of obstruction, disease extent, and clinical condition. Although pooling was consistent with the study’s comparison by operative intent, it may obscure variation in inflammatory-index associations between bypass types. Procedure-specific associations were not evaluated; therefore, the estimates for the pooled palliative-bypass group should not be assumed to apply equally to each procedure subtype.

Independent external validation of AISI and SIRI is required in populations beyond this single-center cohort, including patients treated in different institutions and clinical settings. Prospective multicenter studies should use standardized blood-sampling protocols and consistently document disease extent, biliary obstruction, infection, performance status, nutritional status, CA19-9, and oncological treatment. Validation should assess whether the observed associations are reproducible within resection and palliative-bypass populations. Before clinical implementation, models incorporating these indices should also demonstrate adequate discrimination and calibration, incremental value beyond established clinical variables, and clinical net benefit.

## 5. Conclusions

AISI and SIRI showed statistical heterogeneity in their associations with overall survival between operative groups. Higher values were associated with increased mortality following palliative bypass, whereas no statistically significant association was observed following pancreatic resection. Given the retrospective, single-center design and incomplete adjustment for relevant clinical factors, these findings are hypothesis-generating. Independent external validation in different populations, with comprehensive clinical adjustment and evaluation of prognostic performance, is required before either index can be recommended for clinical use.

## Figures and Tables

**Figure 1:**
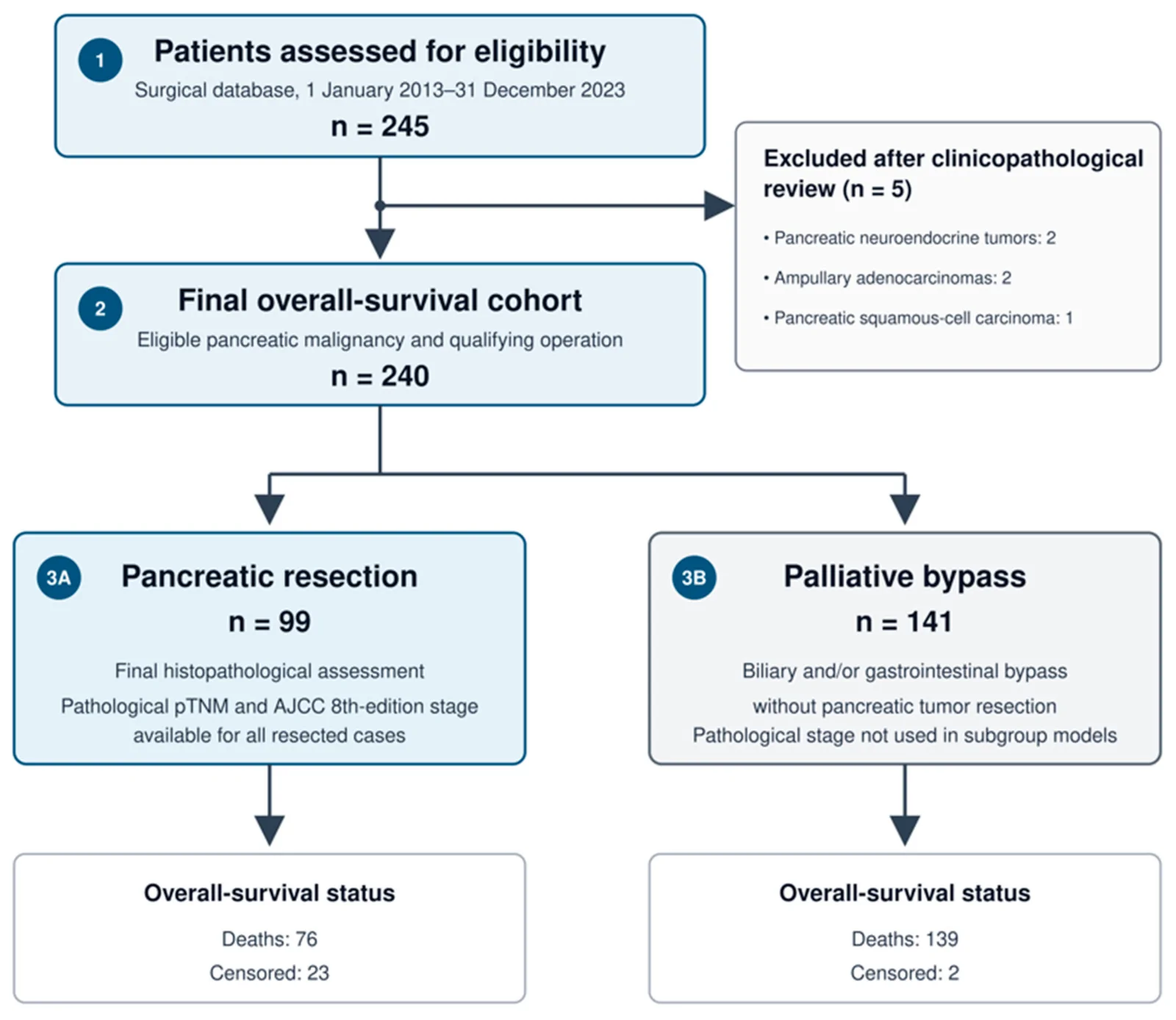
Among the 245 patients assessed for eligibility, five were excluded following clinicopathological review: two patients with pancreatic neuroendocrine tumors, two with ampullary adenocarcinoma, and one with pancreatic squamous-cell carcinoma. The final cohort comprised 240 patients, including 99 who underwent pancreatic resection and 141 who underwent palliative biliary and/or gastrointestinal bypass. During follow-up, 215 deaths occurred, while 25 patients were censored. AJCC, American Joint Committee on Cancer; pTNM, pathological tumor–node–metastasis classification.

**Figure 2:**
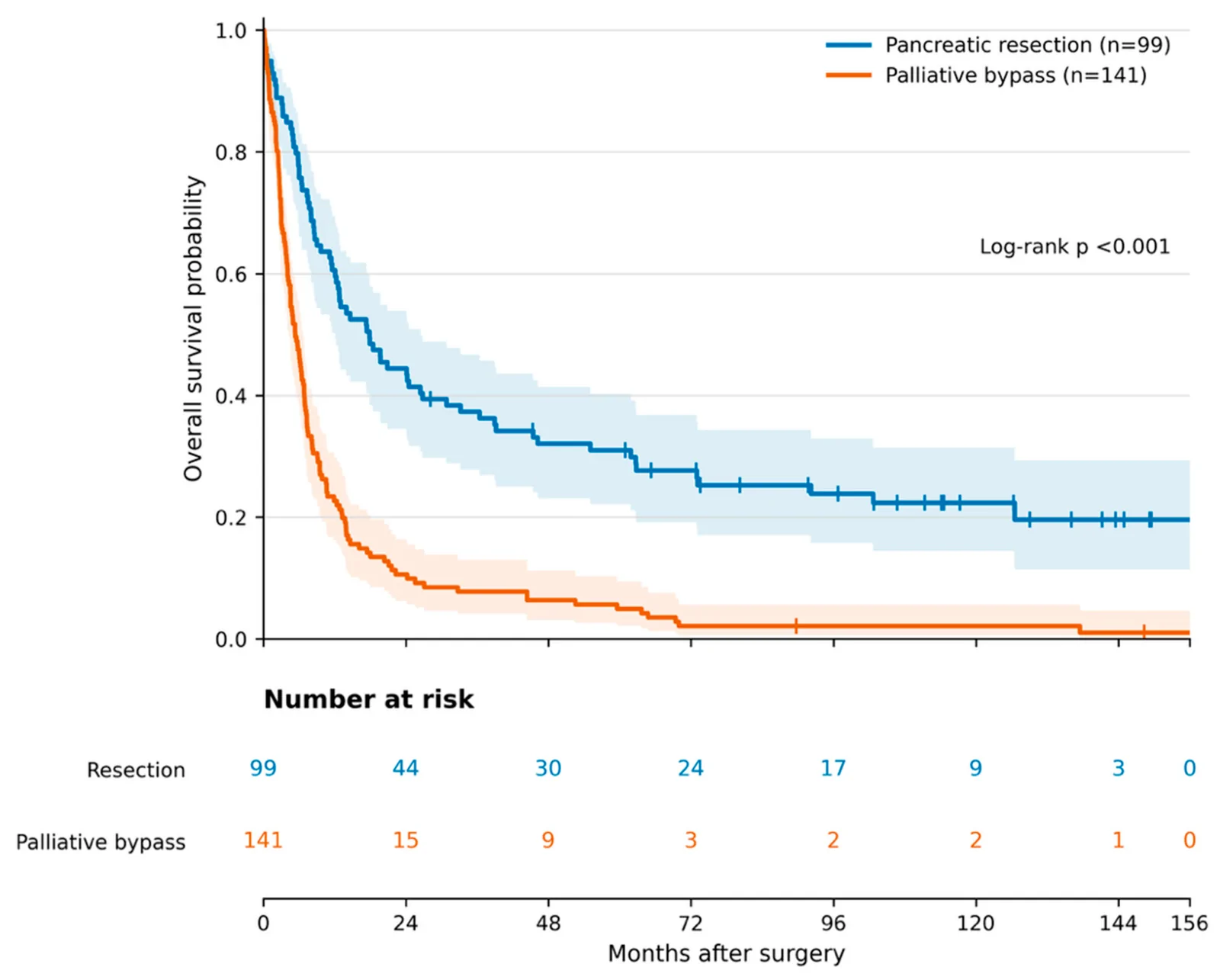
Kaplan–Meier estimates of overall survival according to operative group. Overall survival was measured from the date of surgery until death from any cause or the last documented follow-up. Solid lines represent Kaplan–Meier estimates, shaded areas indicate 95% confidence intervals, and vertical tick marks denote censored observations. The lower panel presents the number of patients at risk. The reported *p* value was calculated using the log-rank test.

**Figure 3:**
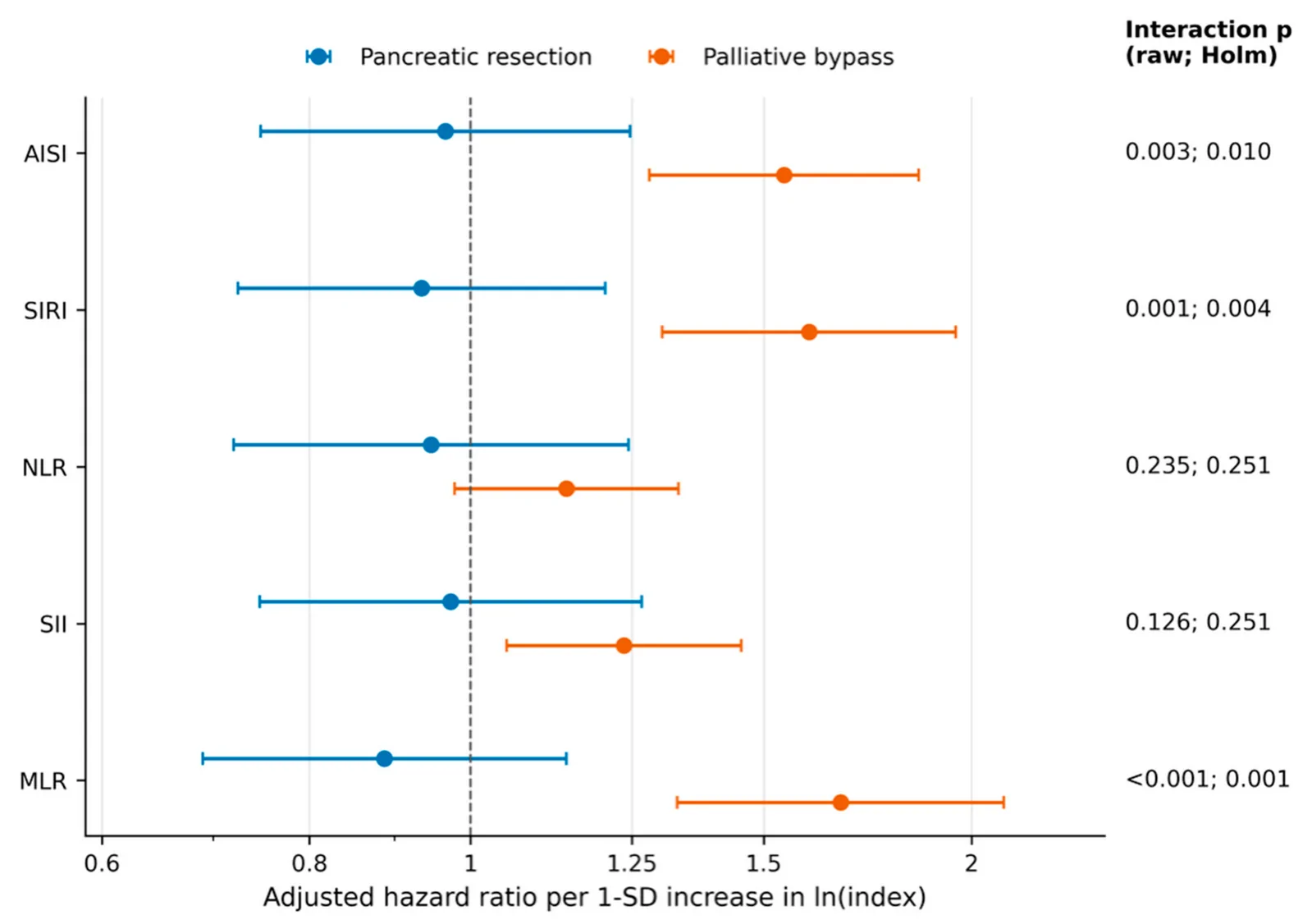
Adjusted associations between systemic inflammatory indices and overall survival according to operative group. Points and horizontal bars represent adjusted hazard ratios and 95% confidence intervals per 1 pooled SD increase in the natural logarithm of each index. Each index was standardized using the combined cohort, so the same numerical increment applies to both operative groups. Hazard ratios are displayed on a logarithmic scale, and the dashed vertical line indicates the null value of 1.0. Models were stratified by operative group, allowed group-specific covariate effects, and included resection-specific AJCC-stage adjustment. The right-hand column presents the raw and Holm-adjusted likelihood-ratio interaction *p* values, respectively.

**Table 1:** Baseline clinical and laboratory characteristics according to operative group.

Characteristic	Overall (*n* = 240)	Pancreatic Resection (*n* = 99)	Palliative Bypass (*n* = 141)
Demographic and clinical characteristics			
Age, years	66.0 (61.0–73.0)	65.0 (58.5–68.0)	69.0 (62.0–76.0)
Male sex	137 (57.1)	51 (51.5)	86 (61.0)
Emergency admission	60 (25.0)	7 (7.1)	53 (37.6)
Diabetes mellitus	79 (32.9)	36 (36.4)	43 (30.5)
Year of cohort entry	2016 (2014–2019)	2016 (2014–2019)	2016 (2015–2019)
Local invasion present, *n*/N (%)	167/213 (78.4)	54/78 (69.2)	113/135 (83.7)
Laboratory characteristics			
Total bilirubin mg/dL	5.78 (0.88–13.11); *n* = 236	1.01 (0.60–7.45); *n* = 98	9.47 (3.22–15.00); *n* = 138
White blood cell count ×10^9^/L	8.00 (6.38–10.50)	7.57 (6.10–10.34)	8.43 (6.50–10.80)
Neutrophil count ×10^9^/L	5.47 (4.08–7.88)	4.93 (3.79–6.27)	5.93 (4.47–8.40)
Lymphocyte count ×10^9^/L	1.41 (1.00–2.00)	1.60 (1.19–2.15)	1.28 (0.95–1.80)
Monocyte count ×10^9^/L	0.62 (0.49–0.85); *n* = 232	0.60 (0.44–0.78); *n* = 96	0.66 (0.50–0.90); *n* = 136
Platelet count ×10^9^/L	252 (198–302); *n* = 239	258 (215–298)	233 (194–308); *n* = 140
Systemic inflammatory indices			
NLR	4.12 (2.53–5.91); *n* = 238	3.10 (2.00–4.97); *n* = 98	4.60 (3.14–7.38); *n* = 140
SII	947 (558–1710); *n* = 237	789 (509–1273); *n* = 98	1052 (642–1903); *n* = 139
MLR	0.44 (0.30–0.70); *n* = 231	0.37 (0.27–0.55); *n* = 95	0.50 (0.33–0.76); *n* = 136
SIRI	2.31 (1.34–5.06); *n* = 231	1.80 (1.08–3.09); *n* = 95	2.70 (1.53–6.43); *n* = 136
AISI	590 (294–1263); *n* = 231	453 (242–830); *n* = 95	636 (330–1546); *n* = 136

Values are presented as median (interquartile range) or *n* (%). Where data were incomplete, the available sample size or denominator is reported. Total bilirubin is expressed in mg/dL, and absolute blood-cell counts are expressed in ×10^9^/L. NLR and MLR are dimensionless ratios; SII, SIRI, and AISI are reported on their calculated numerical scales using blood-cell counts expressed in ×10^9^/L. Inflammatory indices were recalculated directly from their component blood-cell counts. Between-group hypothesis testing was not performed because these comparisons were descriptive and operative allocation was neither randomized nor intended to estimate a treatment effect. AISI, aggregate index of systemic inflammation; IQR, interquartile range; MLR, monocyte-to-lymphocyte ratio; NLR, neutrophil-to-lymphocyte ratio; SII, systemic immune-inflammation index; SIRI, systemic inflammation response index.

**Table 2:** Operative and pathological characteristics of the pancreatic-resection group.

Characteristic	Pancreatic Resection (*n* = 99)
Type of pancreatic resection	
Pancreaticoduodenectomy	74 (74.7)
Distal or subtotal pancreatectomy	23 (23.2)
Total pancreatectomy	1 (1.0)
Duodenum-preserving pancreatic head resection	1 (1.0)
Open surgical approach	99 (100.0)
Splenectomy	16/97 (16.5)
Pathological T category	
pT1	3 (3.0)
pT2	29 (29.3)
pT3	62 (62.6)
pT4	5 (5.1)
Pathological N category	
pN0	38 (38.4)
pN1	43 (43.4)
pN2	18 (18.2)
Distant-metastasis category	
M0	91 (91.9)
M1	8 (8.1)
AJCC eighth-edition stage reconstructed from pT, pN, and M	
Stage IA	3 (3.0)
Stage IB	17 (17.2)
Stage IIA	18 (18.2)
Stage IIB	37 (37.4)
Stage III	16 (16.2)
Stage IV	8 (8.1)
Histological grade	
Moderately differentiated, G2	25 (25.3)
Poorly differentiated, G3	5 (5.1)
Missing or not recorded	69 (69.7)
Total hospital stay, days	14 (11–18)
Postoperative hospital stay, days	12 (9–15)
Intensive care unit stay, days	2 (0–4)
Postoperative death, as recorded	7 (7.1)
Reintervention	10 (10.1)

Values are presented as *n* (%), *n*/N (%), or median (interquartile range). AJCC stage was reconstructed from the verified pathological T and N categories and documented M category according to the eighth edition of the American Joint Committee on Cancer staging system. AJCC, American Joint Committee on Cancer; pT, pathological tumor category; pN, pathological nodal category; M, distant-metastasis category.

**Table 3:** Adjusted operative-group-specific associations between systemic inflammatory indices and overall survival.

Index	*n*/Events	Resection Adjusted HR (95% CI)	*p*	Palliative-Bypass Adjusted HR (95% CI)	*p*	Interaction *p*	Holm-Adjusted Interaction *p*
AISI	227/206	0.97 (0.75–1.25)	0.788	1.54 (1.28–1.86)	<0.001	0.003	0.010
SIRI	227/206	0.93 (0.72–1.20)	0.600	1.60 (1.30–1.96)	<0.001	0.001	0.004
NLR	234/210	0.95 (0.72–1.24)	0.694	1.14 (0.98–1.33)	0.093	0.235	0.251
SII	233/210	0.97 (0.75–1.27)	0.838	1.24 (1.05–1.45)	0.010	0.126	0.251
MLR	227/206	0.89 (0.69–1.14)	0.353	1.67 (1.33–2.09)	<0.001	<0.001	0.001

**Table 4:** Apparent discrimination and robustness analyses for the systemic inflammatory indices.

**Panel A.** Apparent changes in Harrell’s C-index after adding each index.						
**Index**	**Resection Base C-Index**	**Resection + Index**	**ΔC**	**Palliative Base C-Index**	**Palliative + Index**	**ΔC**
AISI	0.672	0.677	0.005	0.570	0.653	0.083
SIRI	0.672	0.679	0.007	0.570	0.643	0.072
NLR	0.684	0.689	0.005	0.574	0.612	0.038
SII	0.684	0.687	0.003	0.575	0.630	0.055
MLR	0.672	0.676	0.004	0.570	0.628	0.058
**Panel B.** Resection-stage sensitivity analyses for AISI and SIRI.						
**Index**	**Stage Specification**		***n*** **/Events**	**Adjusted HR (95% CI)**	**Raw *p***	**Holm-Adjusted *p***
AISI	AJCC stage entered as an ordinal variable		94/74	1.03 (0.79–1.34)	0.837	1.000
AISI	pT4, node-positive status, and M1 entered separately		94/74	1.00 (0.76–1.33)	0.974	1.000
AISI	Restricted to AJCC stages I–III		88/68	0.85 (0.64–1.14)	0.278	0.826
SIRI	AJCC stage entered as an ordinal variable		94/74	0.99 (0.76–1.28)	0.924	1.000
SIRI	Pathological T4, N+, and M1 entered separately		94/74	0.97 (0.74–1.28)	0.829	1.000
SIRI	Restricted to AJCC stages I–III		88/68	0.85 (0.65–1.11)	0.239	0.826
**Panel C.** Robustness analyses of the operative-group interaction.						
**Index**	**Analysis**	***n*** **/Events**	**Resection Adjusted HR (95% CI)**	**Palliative-Bypass Adjusted HR (95% CI)**	**Interaction *p***	**Holm-Adjusted Interaction *p***
AISI	Expanded group-specific adjustment	227/206	0.98 (0.75–1.28)	1.51 (1.25–1.83)	0.009	0.027
SIRI	Expanded group-specific adjustment	227/206	0.94 (0.72–1.23)	1.57 (1.28–1.92)	0.003	0.010
AISI	30-day landmark analysis	209/188	0.98 (0.76–1.27)	1.55 (1.27–1.89)	0.006	0.017
SIRI	30-day landmark analysis	209/188	0.95 (0.73–1.23)	1.55 (1.24–1.92)	0.004	0.016

C-index estimates and their changes are exploratory apparent estimates calculated in the same samples used to fit the models. Base-model and index-augmented C-indices were calculated within the same index-specific complete-case sample. No bootstrap optimism correction, calibration assessment, or external validation was performed. These estimates may overstate performance in new patients and do not establish clinical utility. Expanded adjustment additionally incorporated emergency admission and local-invasion status, including a missing-category indicator. The 30-day landmark analysis excluded patients who died within 30 days after surgery and reset time zero at postoperative day 30. Holm correction was applied across the five marker-specific tests within each analysis family. AJCC, American Joint Committee on Cancer; AISI, aggregate index of systemic inflammation; MLR, monocyte-to-lymphocyte ratio; N+, pathological lymph-node involvement; NLR, neutrophil-to-lymphocyte ratio; SII, systemic immune-inflammation index; SIRI, systemic inflammation response index (Figure 3).

## Data Availability

The datasets generated and/or analyzed during the current study are available from the corresponding author upon reasonable request, subject to institutional and ethical restrictions protecting patient confidentiality.

## Supplementary Materials

The following supporting information can be downloaded at: https://www.mdpi.com/article/10.3390/diseases14100358/s1, Table S1: Missingness and analytic availability of variables included in the study analyses; Table S2: Pairwise Spearman rank correlations among the recalculated systemic inflammatory indices in the overall cohort; Table S3: Proportional-hazards and functional-form diagnostics for the adjusted marker-specific Cox models; Figure S1: Spearman rank-correlation heat map of the recalculated systemic inflammatory indices in the overall analytical cohort; Figure S2: Restricted cubic spline assessment of the association between the preoperative neutrophil-to-lymphocyte ratio and overall mortality following palliative bypass.

## Abbreviations

The following abbreviations are used in this manuscript:

**Abbreviation**	**Definition**
AISI	Aggregate index of systemic inflammation
AJCC	American Joint Committee on Cancer
CA19-9	Carbohydrate antigen 19-9
CCL2	C-C motif chemokine ligand 2
CCR2	C-C chemokine receptor type 2
CI	Confidence interval
FOLFIRINOX	Fluorouracil, leucovorin, irinotecan, and oxaliplatin
HR	Hazard ratio
IQR	Interquartile range
LMR	Lymphocyte-to-monocyte ratio
MLR	Monocyte-to-lymphocyte ratio
NLR	Neutrophil-to-lymphocyte ratio
OS	Overall survival
PDAC	Pancreatic ductal adenocarcinoma
PLR	Platelet-to-lymphocyte ratio
pTNM	Pathological tumor–node–metastasis classification
SD	Standard deviation
SII	Systemic immune-inflammation index
SIRI	Systemic inflammation response index
STROBE	Strengthening the Reporting of Observational Studies in Epidemiology
TNM	Tumor–node–metastasis classification
