# Supplementary material for: Differential Prognostic Associations of Monocyte-Containing and Traditional Systemic Inflammatory Indices with Overall Survival After Resection or Palliative Bypass for Pancreatic Cancer: A Retrospective Cohort Study
Source: Diseases. 2026 Sep 28;14(10):358. doi: 10.3390/diseases14100358 (PMC13703576; doi:10.3390/diseases14100358)
Supplement: Supplementary file 1 [file diseases-14-00358-s001.zip › diseases-4535002-supplementary.pdf]

Table S1. Missingness and analytic availability of variables included in the study analyses.

Variable	Overall applicable population	Pancreatic resection	Palliative bypass
**Clinical and analytical covariates**			
**Age**	0/240 (0.0)	0/99 (0.0)	0/141 (0.0)
**Sex**	0/240 (0.0)	0/99 (0.0)	0/141 (0.0)
**Emergency admission**	0/240 (0.0)	0/99 (0.0)	0/141 (0.0)
**Calendar year of cohort entry**	0/240 (0.0)	0/99 (0.0)	0/141 (0.0)
**Total bilirubin**	4/240 (1.7)	1/99 (1.0)	3/141 (2.1)
**Neutrophil count**	0/240 (0.0)	0/99 (0.0)	0/141 (0.0)
**Lymphocyte count**	0/240 (0.0)	0/99 (0.0)	0/141 (0.0)
**Monocyte count**	8/240 (3.3)	3/99 (3.0)	5/141 (3.5)
**Platelet count**	1/240 (0.4)	0/99 (0.0)	1/141 (0.7)
**Local invasion status**	27/240 (11.3)	21/99 (21.2)	6/141 (4.3)
**Tumor-staging variables**			
**Pathological T (pT) category**	0/99 (0.0)	0/99 (0.0)	Not applicable
**Pathological N (pN) category**	0/99 (0.0)	0/99 (0.0)	Not applicable
**Documented distant-metastasis (M) category**	0/99 (0.0)	0/99 (0.0)	Not applicable
**AJCC eighth-edition stage**	0/99 (0.0)	0/99 (0.0)	Not applicable
**Histological grade**	69/99 (69.7)	69/99 (69.7)	Not applicable
**Recalculated inflammatory indices**			
**AISI unavailable for analysis**	9/240 (3.8)	4/99 (4.0)	5/141 (3.5)
**SIRI unavailable for analysis**	9/240 (3.8)	4/99 (4.0)	5/141 (3.5)
**NLR unavailable for analysis**	2/240 (0.8)	1/99 (1.0)	1/141 (0.7)
**SII unavailable for analysis**	3/240 (1.3)	1/99 (1.0)	2/141 (1.4)
**MLR unavailable for analysis**	9/240 (3.8)	4/99 (4.0)	5/141 (3.5)

Table note: Tumor-staging variables were applicable only to the pancreatic-resection group. The pT and pN categories were obtained from final histopathological assessment, whereas M status was determined from the available pathological, operative, and radiological documentation..

Table S2. Pairwise Spearman rank correlations among the recalculated systemic inflammatory indices in the overall cohort.

Inflammatory index	AISI	SIRI	NLR	SII	MLR
**AISI**	1.00	0.94	0.80	0.88	0.78
**SIRI**	0.94	1.00	0.86	0.80	0.87
**NLR**	0.80	0.86	1.00	0.88	0.72
**SII**	0.88	0.80	0.88	1.00	0.60
**MLR**	0.78	0.87	0.72	0.60	1.00

Table note: Values are Spearman rank-correlation coefficients (ρ) calculated using pairwise complete observations from the overall analytical cohort. All off-diagonal correlations had $p < 0.001$. The correlations were descriptive and were not adjusted for multiplicity. AISI, aggregate index of systemic inflammation; MLR, monocyte-to-lymphocyte ratio; NLR, neutrophil-to-lymphocyte ratio; SII, systemic immune-inflammation index; SIRI, systemic inflammation response index.

Table S3. Proportional-hazards and functional-form diagnostics for the adjusted marker-specific Cox models.

Operative group	Index	n/events	Index-specific PH test, *p*	Global PH test, *p*	Nonlinearity test, *p*
**Pancreatic resection**	AISI	94/74	0.778	0.626	0.294
**Pancreatic resection**	SIRI	94/74	0.920	0.643	0.347
**Pancreatic resection**	NLR	97/75	0.619	0.698	0.207
**Pancreatic resection**	SII	97/75	0.357	0.619	0.672
**Pancreatic resection**	MLR	94/74	0.845	0.654	0.232
**Palliative bypass**	AISI	133/132	0.568	0.761	0.726
**Palliative bypass**	SIRI	133/132	0.258	0.618	0.902
**Palliative bypass**	NLR	137/135	**0.007**	0.122	**<0.001**
**Palliative bypass**	SII	136/135	0.068	0.428	0.168
**Palliative bypass**	MLR	133/132	0.416	0.784	0.287

Table note The marker-specific proportional-hazards test compared the adjusted model with and without a marker-by-log(1 + follow-up time) interaction term using a likelihood-ratio test. The global proportional-hazards test jointly evaluated corresponding time-interaction terms for all model coefficients. Follow-up time was expressed in days. The global proportional-hazards test jointly evaluated time interactions for all model coefficients. Nonlinearity was assessed by comparing the linear index specification with a restricted cubic spline model using four knots positioned at the 5th, 35th, 65th, and 95th percentiles. Models were adjusted for age, sex, ln(total bilirubin), and calendar year; models restricted to the resection group were additionally adjusted for AJCC stage III–IV versus I–II. Diagnostic *p* values were not adjusted for multiplicity. A small *p* value indicates evidence against the corresponding model assumption. AISI, aggregate index of systemic inflammation; AJCC, American Joint Committee on Cancer; MLR, monocyte-to-lymphocyte ratio; NLR, neutrophil-to-lymphocyte ratio; PH, proportional hazards; SII, systemic immune-inflammation index; SIRI, systemic inflammation response index.

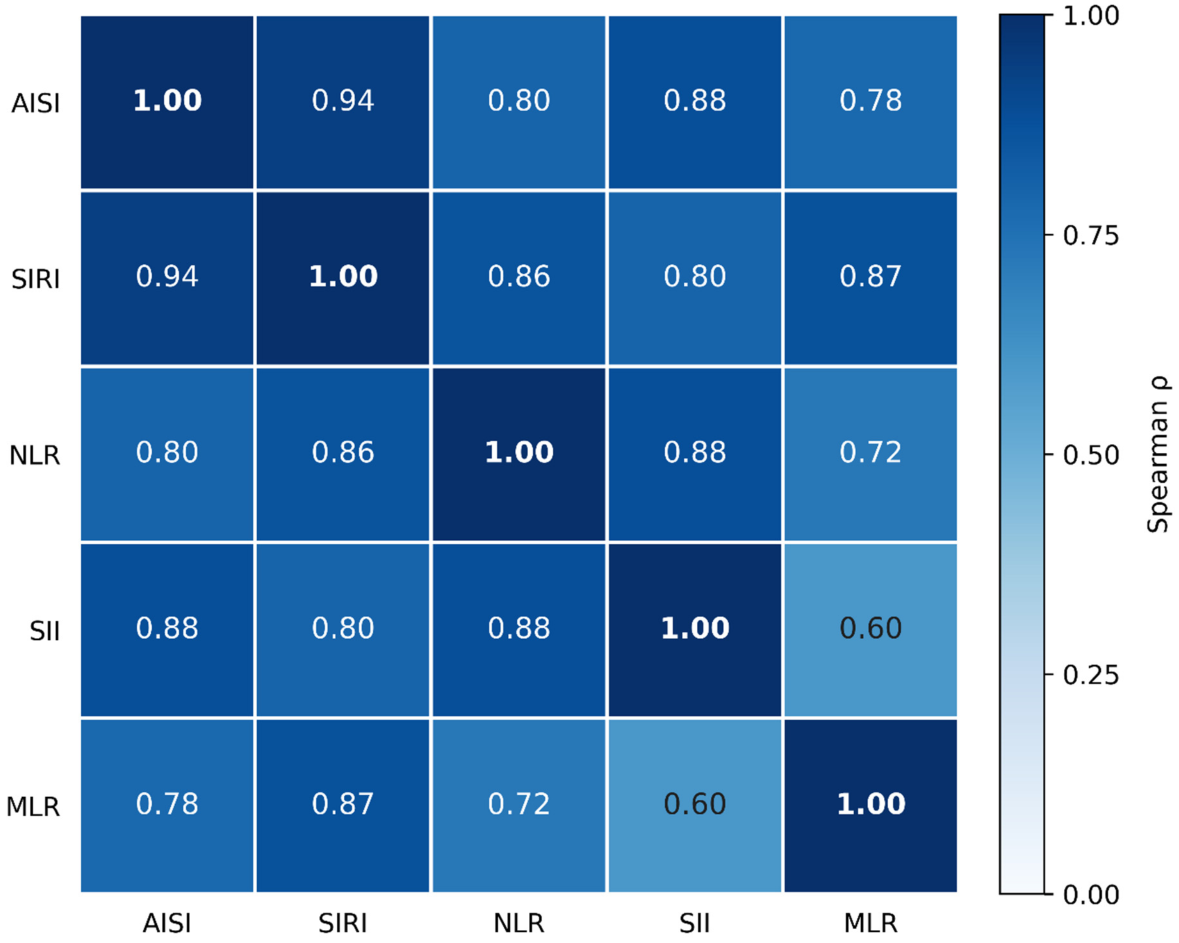

Figure S1. Spearman rank-correlation heat map of the recalculated systemic inflammatory indices in the overall analytical cohort. Each cell displays the Spearman correlation coefficient (ρ), calculated using pairwise complete observations. All off-diagonal correlations had $p < 0.001$. AISI, aggregate index of systemic inflammation; MLR, monocyte-to-lymphocyte ratio; NLR, neutrophil-to-lymphocyte ratio; SII, systemic immune-inflammation index; SIRI, systemic inflammation response index.

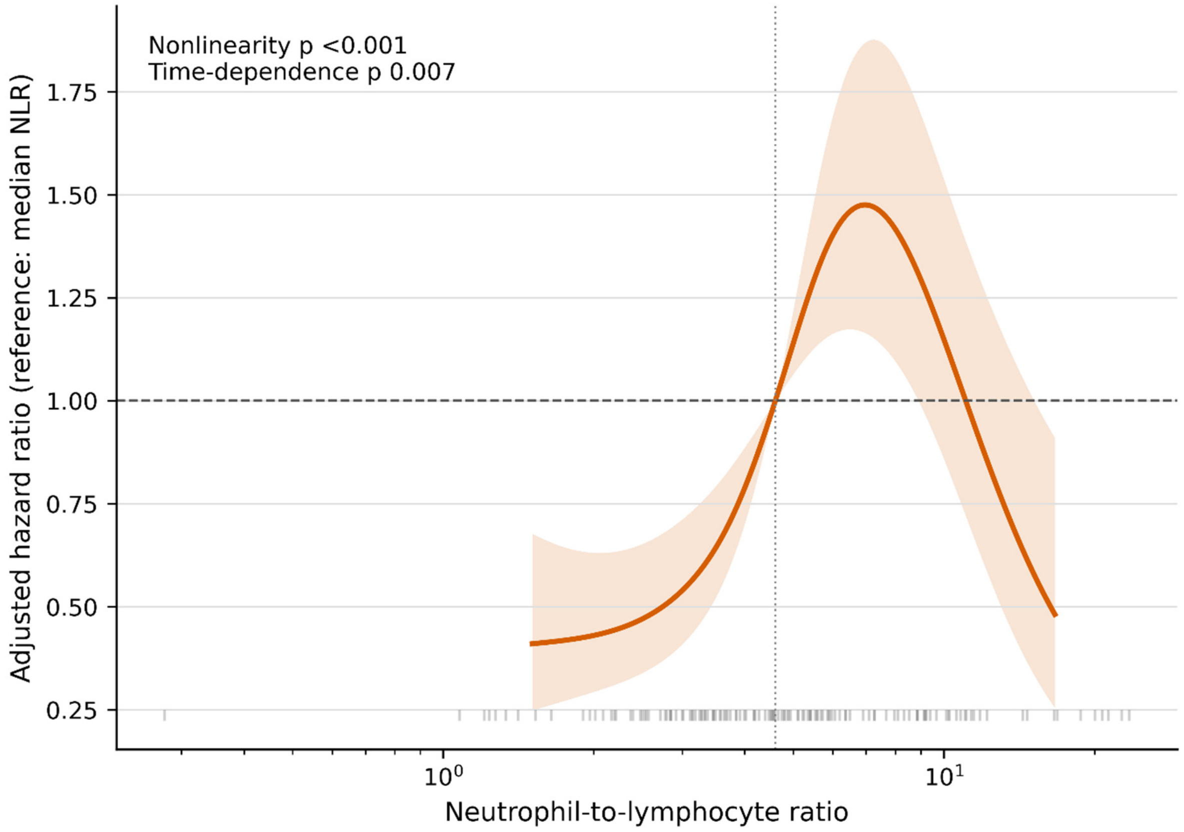

Figure S2. Restricted cubic spline assessment of the association between the preoperative neutrophil-to-lymphocyte ratio and overall mortality following palliative bypass. The solid line represents the adjusted hazard ratio relative to the median NLR, and the shaded area represents the 95% confidence interval. The horizontal dashed line indicates the null value of 1.0, the vertical dotted line indicates the median NLR, and rug marks show the observed NLR distribution. The curve is displayed between the 5th and 95th percentiles. The model included 137 patients and 135 deaths and was adjusted for age, sex, ln(total bilirubin), and calendar year. The association showed evidence of nonlinearity ($p < 0.001$) and time dependence ($p = 0.007$). Because the proportional-hazards assumption was violated, this spline should be interpreted as a functional-form diagnostic rather than as a time-invariant prognostic effect or evidence supporting a clinical NLR cut-off.
